# Quality of life and disease burden in tuberous sclerosis and comparison with the population with idiopathic autism spectrum disorder: an investigation conducted through questionnaires and clinical data collection in the pediatric population

**DOI:** 10.3389/fpsyt.2025.1730160

**Published:** 2026-01-14

**Authors:** Giorgia Sforza, Alessandra Voci, Virginia Tonietti, Giacomo Racioppi, Martina Proietti Checchi, Massimiliano Valeriani, Luigi Mazzone, Mariagrazia Cicala, Leonardo Emberti Gialloreti, Romina Moavero

**Affiliations:** 1 Developmental Neurology Unit, Bambino Gesù Children’s Hospital, Istituto di Ricovero e Cura a Carattere Scientifico (IRCCS), Rome, Italy; 2 Child Neurology and Psychiatry Unit, Systems Medicine Department, Tor Vergata University of Rome, Rome, Italy; 3 Residency School of Pediatrics, Tor Vergata University of Rome, Rome, Italy; 4 Academy of Pediatrics, Bambino Gesù Children’s Hospital, IRCCS, Rome, Italy; 5 Translational Pain Neuroscience and Precision Medicine, Center for Neuroplasticity and Pain (CNAP), Department of Health Science and Technology, School of Medicine, Aalborg University, Aalborg, Denmark; 6 Department of Biomedicine and Prevention, Tor Vergata University of Rome, Rome, Italy

**Keywords:** ASD, burden, children, epilepsy, quality of life, TAND, TSC

## Abstract

**Introduction:**

Tuberous sclerosis complex (TSC) is a rare genetic disease involving major neurological and neuropsychiatric symptoms that can impact quality of life. This study aimed to examine the quality of life and disease burden of a pediatric TSC cohort and compare them with those of a population of individuals with idiopathic autism spectrum disorder (ASD).

**Methods:**

Caregivers completed the Tuberous Sclerosis-Associated Neuropsychiatric Disorders (TAND) Checklist and the Pediatric Quality of Life Inventory™ (PedsQL) Report. To examine quality of life, caregivers also completed the TSC Quality of Life (TSCQoL) questionnaire, which was created specifically for this study to investigate the emotional, economic, and time-related impact of TSC. We recruited 66 individuals with TSC (average age, 9.8 ± 4.5 years) and 63 individuals with idiopathic ASD (8.4 ± 4.4 years).

**Results:**

We found a reduction in quality of life and a significant economic investment in 50% and 55% of TSC patients, respectively. These aspects were significantly more prevalent in individuals with cognitive impairment, ASD, sleep disorders, and epilepsy. Individuals with idiopathic ASD exhibited worse quality of life in the social domain (*p* = 0.004), while the syndromic ASD group demonstrated worse overall quality of life (*p* = 0.041) and experienced a greater loss of school days (*p* = 0.039).

**Discussion:**

Despite its lack of validation, the TSCQoL scale was established as an exploratory tool that consistently highlighted, along with the PedsQL, clinical factors that most impact quality of life. Quality of life was low in the TSC population, and this was strongly correlated with neuropsychiatric manifestations and epilepsy. Through comparison with idiopathic ASD, we observed a greater impact of the syndromic condition on disease burden.

## 1 Introduction

Pediatric genetic disorders are a group of conditions that can have a significant impact on affected individuals and their families. Tuberous sclerosis complex (TSC) is a paradigmatic example of a multisystem disorder with a wide clinical spectrum. TSC is an autosomal dominant genetic disorder caused by mutations in either the *TSC1* or *TSC2* gene. These mutations lead to disruption in the mammalian target of rapamycin (mTOR) signaling pathway, which regulates cell growth, protein synthesis, and metabolism (1). Hyperactivation of mTOR in TSC results in the development of hamartomas, which are benign lesions that can affect multiple organs and systems, including the brain, skin, heart, kidneys, and lungs (2).

TSC affects approximately one in 6,000 individuals in the general population. Clinical expression varies not only across individuals but also within families. Thus, while some individuals experience significant symptoms, many affected individuals exhibit mild or asymptomatic clinical signs and often remain undiagnosed (3).

Most people with TSC exhibit central nervous system involvement driven by altered cortical structure and connectivity (4). Neurological and psychiatric manifestations of TSC include epilepsy and TSC-associated neuropsychiatric disorders (TAND). The term “TAND” was introduced by the Expert Panel of the 2012 International Consensus Conference and encompasses neurodevelopmental conditions such as intellectual disability (ID), autism spectrum disorder (ASD), and attention deficit hyperactivity disorder (ADHD), as well as neuropsychological and behavioral problems (5, 6). These manifestations typically present in an age-related manner, with different characteristics emerging at various developmental stages and exhibiting considerable variability (7). Some TSC patients may experience significant intellectual, developmental, and behavioral disorders, while others lead normal lives.

The complexity of TSC extends well beyond its clinical features. As a lifelong condition, TSC imposes a substantial burden on quality of life (QoL). According to the World Health Organization (WHO), QoL reflects the individual’s perception of their life situation within their cultural and value context, taking into account their aspirations, expectations, and concerns (8). In chronic conditions such as TSC, multisystem issues, epilepsy, cognitive impairment, and behavioral disturbances can significantly compromise daily functioning, limit autonomy and social integration, and increase the stress of both patients and caregivers (9, 10). Moreover, the economic impact of TSC is considerable, encompassing both direct medical costs (such as expenses for therapies, medical evaluations, and specialized treatments) and indirect costs related to caregivers’ productivity loss due to reduced working hours or unemployment (11–13).

The extent and complexity of TSC-related manifestations raise the question of whether the burden linked with psychiatric symptoms in TSC exceeds that of other neurodevelopmental conditions—such as idiopathic ASD—that are not associated with genetic or multisystem organ involvement. Recent studies have shown a reduction in QoL in TSC, particularly in association with drug-resistant epilepsy and comorbid neurodevelopmental disorders, but have mainly focused on adults or mixed adult–pediatric cohorts and have not directly compared TSC with the population with idiopathic neurodevelopmental disorders (10, 12, 14). These considerations underscore the need for a comparative, pediatric-focused study to better delineate the specific burden of TSC versus idiopathic conditions such as ASD.

The primary objective of our study was to evaluate QoL and disease burden in a group of pediatric patients with TSC and analyze their relationship with clinical profile, cognitive level, adaptive skills, and behavioral challenges. The secondary objective was to compare the QoL and disease burden of subjects with ASD secondary to TSC to those with idiopathic ASD in order to identify factors contributing to differential clinical, economic, and social impacts on patients and families.

## 2 Materials and methods

### 2.1 Participants

Our study was conducted at the Child Neurology and Psychiatry Unit of Tor Vergata University Hospital and the Developmental Neurology Unit of Bambino Gesù Children’s Hospital. Written informed consent was obtained from the parents or legal guardians of all participants. The study was approved by the Tor Vergata University Hospital Ethical Committee (approval registration number 103.23).

We recruited 66 individuals with a confirmed diagnosis of TSC, according to established diagnostic criteria (15), aged between 18 months and 18 years [mean, 9.8 ± 4.5; median, 10.2; interquartile range (IQR) = 2.2–17.9]. The group consisted of 39 men (59%) and 27 women (41%). Exclusion criteria included the presence of other acute medical conditions that could affect QoL and poor family compliance in completing the provided questionnaires.

Also, 63 individuals diagnosed with idiopathic ASD, according to the *Diagnostic and Statistical Manual of Mental Disorders*, Fifth Edition (DSM-5) criteria, were enrolled (16). Participants were aged 18 months to 18 years (mean, 8.4 ± 4.4; median, 7.6; IQR = 2.1–17.8) and consisted of 38 men (60%) and 25 women (40%). Exclusion criteria included other neurological or genetic conditions, while common psychiatric ASD comorbidities were retained for clinical representativeness; patients whose families lacked compliance in completing the provided questionnaires were also excluded.

Both groups were classified by most recent cognitive assessments into intellectual functioning ranges using standardized intelligence quotient (IQ)/developmental quotient scores: normal (IQ ≥ 85), borderline (70–84), mild ID (50–69), moderate ID (35–49), and severe ID (<35). Distributions were comparable (TSC: 41% normal IQ, 15% borderline, 18% mild ID, 21% moderate ID, and 5% severe ID; ASD: 38% normal IQ, 16% borderline, 22% mild ID, and 24% moderate ID).

Regarding comorbidities in TSC patients, 53% had a diagnosis of epilepsy, and 76% had a clinical diagnosis of at least one TAND. Cognitive impairment was the most common TAND, affecting 59% of patients. Other frequently encountered TANDs included ASD (48%) and ADHD (21%), while 38% had other TANDs. Considering the ASD population, four patients (6%) had a diagnosis of ADHD, and six patients (10%) had other neuropsychiatric conditions, including language disorder, developmental coordination disorder, behavioral disorder, anxiety and other emotional disorders, pica disorder, and ultra-high-risk state for psychosis.

Complete demographic and clinical details about the TSC population are reported in **Table 1**.

**Table 1:** Characteristics of the TSC population (N = 66) based on previous clinical evaluations, databases, and parent interviews.

Characteristic	Mean ± SD (median; IQR)
Age (years)	9.8 ± 4.5 (10.2, 2.2–17.9)
Characteristic	N (%)
Sex	
Male	39 (59%)
Female	27 (41%)
Genetics	
Pathogenic or likely pathogenic mutation	49 (74%)
TSC1 mutation	11 (17%)
TSC2 mutation	38 (58%)
NMI	12 (18%)
Pending or not performed genetic analysis	5 (8%)
Epilepsy	
Diagnosis of epilepsy (previous or active)	35 (53%)
Pharmacologically treated epilepsy	30 (45%)
Drug-resistant epilepsy	18 (27%)
Surgical treatment for epilepsy	4 (6%)
TAND	
≥1 TAND	50 (76%)
Cognitive impairment	39 (59%)
Borderline functioning	10 (15%)
ID	29 (44%)
ASD	32 (48%)
ADHD	14 (21%)
Other TANDs^*^	25 (38%)
CNS lesions	
SEGA	15 (23%)
SEN at the foramen of Monro	48 (73%)
Systemic manifestations	
Renal manifestations	
Angiomyolipomas or cysts	48 (73%)
Angiomyolipomas or cysts ≥2 cm	8 (12%)
Renal function impairment	4 (6%)
Cardiac manifestations	
Cardiac rhabdomyomas	53 (80%)
Cardiac function impairment	10 (15%)
Ocular manifestations	18 (27%)
Cutaneous manifestations	60 (91%)

NMI, no mutation identified; TAND, TSC-Associated Neuropsychiatric Disorder; ID, intellectual disability; ASD, autism spectrum disorder; ADHD, attention deficit hyperactivity disorder; CNS, central nervous system; SEGA, subependymal giant cell astrocytoma; SEN, subependymal nodule; SD, standard deviation; IQR, interquartile range; N, number of observations.

^*^Including language disorder, learning disorder, anxiety disorder, behavioral disorder, obsessive compulsive disorder, oppositional defiant disorder, tic disorder, and sleep disorder.

### 2.2 Procedures

Patients diagnosed with TSC or idiopathic ASD were enrolled if they had previously undergone cognitive and behavioral evaluations. This was conducted to ensure that the populations were homogeneous in terms of cognitive impairment and neuropsychiatric comorbidities. Child neurologists and psychiatrists with expertise in both TSC and neurodevelopment then conducted clinical and anamnestic evaluations. These evaluations included reviewing medical databases and interviewing caregivers to assess systemic, neuropsychiatric, and neurological manifestations.

Based on the most recent available cognitive assessments, we classified patients into intellectual functioning ranges. Among the TSC patients, we also identified those who met the criteria for a comorbid ASD diagnosis. ASD severity levels were not available and homogeneous for all enrolled children, as a recent and complete Autism Diagnostic Observation Schedule (ADOS) assessment was missing for some ASD participants. To avoid introducing selection bias and losing cases with otherwise complete clinical and cognitive data, we prioritized intellectual functioning as the primary severity measure, which showed comparable distributions across groups.

The primary caregivers of patients with TSC or idiopathic ASD completed a set of standardized instruments under the supervision of a psychologist expert in the field. These instruments are validated in the Italian language and applicable to subjects of preschool age to adolescence (17–21). The instruments included the Child Behavior Checklist (CBCL) (22), the Adaptive Behavior Assessment System—Second Edition (ABAS-II) (23), the TAND Checklist (24), and the Pediatric Quality of Life Inventory™ (PedsQL) (25). Finally, the caregivers filled out a study-specific questionnaire, the TSC Quality of Life (TSCQoL) questionnaire, designed to better investigate the impact and degree of emotional, financial, and time investment related to TSC. This supervised administration ensured complete data collection, as any missing items were immediately identified and re-presented to parents or caregivers for completion, resulting in no missing data.

We analyzed the results of the questionnaires within the TSC group and examined the relationships between clinical characteristics, cognitive level, adaptive functioning, behavioral characteristics, and QoL outcomes, as assessed using the PedsQL and TSCQoL. Additionally, we compared the results of the TSC group with those of the idiopathic ASD group and those of patients with ASD secondary to TSC to explore differences in behavior, adaptive functioning, and QoL.

### 2.3 Neuropsychological questionnaires

#### 2.3.1 Child Behavior Checklist

The CBCL is a questionnaire used to evaluate the behavior of children and adolescents. Based on age, it exists in two formats (1.5–5 and 6–18 years) and examines the presence of externalizing and internalizing problems, which are broken down into several subscales. For our analysis, we considered the scores for *internalizing problems* and *externalizing problems*, as well as *total problems*, since these are summary measures assessed using both formats of the test (22). According to the normative data, we considered a T-score < 60 as non-clinical, a T-score between 60 and 64 as a borderline range, and a T-score ≥ 65 as clinically significant.

#### 2.3.2 Adaptive Behavior Assessment System—Second Edition

The ABAS-II is a behavior rating scale that measures daily living skills across various life domains (23). Specifically, it examines 10 adaptive areas that can be attributed to three domains: the *Conceptual Adaptive Domain* (CAD) includes areas related to communication, preschool/school skills, and self-control; the *Social Adaptive Domain* (SAD) involves play/leisure and socialization abilities; and the *Practical Adaptive Domain* (PAD) concerns self-care, home/school life, use of the environment, and health and safety. The sum of the scores determines the *General Adaptive Composite* (GAC) index, which provides a global score related to adaptive functioning and is expressed as a quotient. An adequate level of functioning at or above average corresponds to a quotient of at least 90. In our study, we used a questionnaire designed for parents or caregivers to complete the assessment.

#### 2.3.3 TAND Checklist

The *TAND Checklist* is a structured screening interview designed to identify neuropsychiatric symptoms and assess multiple domains in individuals with TSC, including behavioral difficulties, psychiatric disorders, intellectual abilities, academic skills, neuropsychological abilities, and psychosocial functioning (24). Administered by a specialist to caregivers, it provides a dimensional exploration of symptoms, regardless of specific diagnostic categorization.

The checklist is conducted as an interview with either a parent or, when possible, the person directly. This tool does not include a rating scale to assess the severity of each symptom because most questions require only a “yes” (a given difficulty occurs) or “no” (it does not occur) response (26).

The TAND Checklist was administered only to patients with TSC to provide a comprehensive characterization of this population. Data related to basic adaptive functioning, such as language, self-care, and motility (question 2), were collected. Additionally, the behavioral and neuropsychological sections (questions 3 and 7, respectively) were used to examine neuropsychiatric symptoms independently of DSM-5 diagnostic criteria. Additional considered aspects were caregiver-reported family stress and perceived need for further clinical evaluation or support (question 8).

### 2.4 QoL questionnaires

#### 2.4.1 Pediatric Quality of Life Inventory

We used the PedsQL to assess the QoL of patients with TSC and idiopathic ASD. The PedsQL is a questionnaire designed to measure health-related QoL in children and adolescents, both healthy and those with acute or chronic health conditions (25). The PedsQL questionnaire consists of four subscales (*physical functioning*, *emotional functioning*, *social functioning*, and *school functioning*) designed to measure the fundamental health dimensions outlined by the WHO, along with a total summary scale. We utilized the standardization proposed by Huang (2009) to interpret the obtained scores, which stratifies the results into levels of QoL impairment (27).

#### 2.4.2 TSC Quality of Life

The TSCQoL, a questionnaire tailored to individuals with TSC, was designed to evaluate symptom burden and the emotional, economic, and time investments related to the disease. The TSCQoL was created as an exploratory and condition-specific tool and has not yet been validated. The PedsQL was used as the primary QoL measure for all between-group comparisons, whereas the TSCQoL was included as a supplementary instrument developed to capture TSC-relevant domains that are underrepresented in generic tools like the PedsQL. The first part of the questionnaire gave families the opportunity to express their level of concern regarding various TSC-related issues. Beyond TSC-specific domains that provide greater insight into the multisystem nature of the disease, in the second part of the questionnaire, the TSCQoL also explores family investment (economic and time burden), as well as days of work lost by caregivers and days of school lost by patients, which are areas not specifically captured by the PedsQL. For the TSC versus ASD comparison using the TSCQoL, only these non-TSC-specific items were administered to the ASD population to ensure applicability and minimize bias. A detailed explanation of the questionnaire items is provided in **Supplementary File 1**.

### 2.5 Statistical analysis

Descriptive statistics are presented as the mean ± standard deviation (SD), median, and IQR for continuous variables, and as percentages and the number of observations for categorical variables. To compare the TSC and ASD samples, independent-samples t-tests were used for group-to-group comparisons of continuous variables (PedsQL scores). Pearson’s chi-squared test was used for categorical variables (dichotomized TSCQoL scores). For the TSC sample, non-parametric Mann–Whitney tests were used to assess associations between categorical clinical features/neuropsychiatric symptoms and QoL (PedsQL) and family commitment or concern (TSCQoL). An alpha level of 0.05 was used for all statistical analyses. When performing multiple comparisons, the *p*-value was adjusted using the Bonferroni correction. To keep the family-wise error rate at less than 0.05, the alpha level was set at 0.002, 0.004 (for ABAS-II), or 0.005 (for PedsQL), according to the different number of comparisons. The Kruskal–Wallis test was employed to compare QoL across different cognitive levels (with Dunn’s test for *post-hoc* analyses). Bivariate Spearman’s correlation was used to evaluate the relationship between adaptive skills (ABAS-II) and behavioral problems (CBCL), and QoL (PedsQL). The association between family commitment (TSCQoL), dichotomized through the identification of cut-off values, and clinical presentation using univariate logistic regression was analyzed. Subsequently, only the statistically significant variables were included in a multivariable logistic regression to assess each variable’s adjusted impact. All statistical analyses were performed using SPSS v26.0 (IBM Corp.).

## 3 Results

### 3.1 Analysis of TSC population

#### 3.1.1 Neuropsychological questionnaires

Analysis of adaptive functioning using the ABAS-II test revealed that 76% (n = 50) of patients with TSC had a GAC score below the normal level. Of those patients, 69% (n = 46) scored below the normal level in the CAD, 67% (n = 44) in the SAD, and 75% (n = 49) in the PAD.

Of the TSC population, 18% (n = 12) had clinically significant total scores on the CBCL questionnaire, while another 18% (n = 12) had borderline scores. Concerning internalizing disorders, 18% (n = 12) of the population had clinically significant scores, and 8% (n = 5) had borderline scores. For externalizing disorders, 14% (n = 9) had clinically significant scores, and 9% (n = 6) had borderline scores.

#### 3.1.2 TAND Checklist

Of the data collected using the TAND Checklist, 53% of patients (n = 35) were reported to have a deficit in language skills. Regarding self-care, 60% (n = 40) required caregiver support. Motility appeared to be the least impaired parameter, with 20% (n = 13) requiring support.

The most frequently reported concerns were difficulties with attention (73%, n = 48), deficits in executive functions (59%, n = 39), and hyperactivity-impulsivity (50%, n = 33). Forty-eight percent of the sample (n = 32) exhibited angry outbursts, and aggression or self-harm was present in 32% (n = 21). Additionally, rigid and repetitive behaviors (45%, n = 30) and difficulties in social relationships (42%, n = 28) were common. Mood alterations and anxiety symptoms were observed in 35% (n = 23) and 33% (n = 22) of patients, respectively. Lastly, 24% of parents (n = 16) reported the presence of an eating disorder.

Additional findings included family stress (18%, n = 12) and parental stress (35%, n = 23). Furthermore, 32% of families (n = 21) expressed the need for further evaluation or support to address their children’s difficulties.

#### 3.1.3 PedsQL

Fifty percent of TSC patients (n = 33) exhibited impaired functioning on the PedsQL total scale. Specifically, 54% (n = 36) reported difficulties with health and physical activity, 39% (n = 26) with emotional states, 43% (n = 28) with social life, and 39% (n = 25) with school functioning. The distribution of PedsQL scores and descriptive statistics in TSC patients is presented in **Supplementary Table 1**.

#### 3.1.4 TSCQoL

Sixty-five percent (n = 43) of the families reported significant worry about their child’s current condition, and 68% (n = 45) expressed major concerns about future outcomes. Families were particularly troubled by current and future neuropsychiatric, neurological, and renal conditions.

In terms of economic and time investment, 55% of families (n = 36) reported spending over €100 per month on TSC-related expenses. Additionally, 46% (n = 30) spent at least 4 to 5 hours per week managing their children’s needs. In 46% (n = 30) of cases, parents missed three or more days of work per month, and 38% (n = 25) of children missed three or more days of school. Details are provided in **Supplementary Table 2**.

### 3.2 Association between quality of life and clinical manifestations in the TSC population

The clinical manifestations of TSC patients and some specific items from the TAND Checklist, the ABAS-II, and the CBCL were then related to the results of the PedsQL and TSCQoL.

#### 3.2.1 PedsQL

We analyzed the distribution of PedsQL results among groups of patients with various TSC-related manifestations (**Table 2**) and found that active epilepsy is significantly related to the total PedsQL functioning score (U = 198.5, *p* < 0.001) as well as the physical (U = 199, *p* < 0.001), social (U = 214.5, *p* < 0.001), and school (U = 213.5, *p* < 0.001) subscale scores. The presence of ASD significantly impacts the overall QoL (U = 162.5, *p* < 0.001) and all subareas of functioning explored by the test. Sleep disturbances were also associated with reduced QoL, as evidenced by the total score (U = 236, *p* < 0.001) and the physical (U = 225, *p* < 0.001) and school (U = 175.5, *p* < 0.001) subscale scores.

**Table 2:** Association between clinical features (based on previous clinical evaluations, databases, and parent interviews) and PedsQL in TSC patients.

TSC	Statistics	PedsQL Total	PedsQL Health and physical activity	PedsQL Emotional states	PedsQL Social life	PedsQL School
ASD	Mann–Whitney U	162.5	160.5	262	177.5	211.5
	*p*-Value	**<0.001**	**<0.001**	**<0.001**	**<0.001**	**<0.001**
ADHD	Mann–Whitney U	262.5	313.5	288.5	319	198
	*p*-Value	0.111	0.426	0.233	0.476	0.025
Sleep disorders	Mann–Whitney U	236	225	320.5	337	175.5
	*p*-Value	**<0.001**	**<0.001**	0.014	0.024	**<0.001**
Other neuropsychiatric manifestations	Mann–Whitney U	369.5	447.5	443.5	443.5	305.5
	*p*-Value	0.059	0.388	0.359	0.034	**0.015**
Epilepsy	Mann–Whitney U	198.5	199	199	214.5	213.5
	*p*-Value	**<0.001**	**<0.001**	0.005	**<0.001**	**<0.001**
Drug-resistant epilepsy	Mann–Whitney U	120	91	145.5	132	119
	*p*-Value	0.408	0.670	0.958	0.678	0.539
SEGA	Mann–Whitney U	271.5	288	257.5	254	303
	*p*-Value	0.128	0.206	0.079	0.069	0.412
Renal angiomyolipomas or cysts	Mann–Whitney U	316	352	350	349	267.5
	*p*-Value	0.095	0.247	0.235	0.227	0.093
Renal function impairment	Mann–Whitney U	118	102.5	112	145.5	106.5
	*p*-Value	0.872	0.562	0.745	0.559	0.700
Cardiac rhabdomyomas	Mann–Whitney U	270.5	261	352	270.5	239
	*p*-Value	0.233	0.176	0.903	0.228	0.120
Cardiac function impairment	Mann–Whitney U	211	235.5	230.5	224	178
	*p*-Value	0.217	0.424	0.373	0.312	0.177

TSC, tuberous sclerosis complex; ASD, autism spectrum disorder; ADHD, attention deficit hyperactivity disorder; SEGA, subependymal giant cell astrocytoma; PedsQL, Pediatric Quality of Life Inventory.

p-Value adjusted after Bonferroni correction at 0.002.

Bold values indicate statistically significant results.

The presence of drug-resistant epilepsy, ADHD, subependymal giant cell astrocytomas (SEGAs), renal angiomyolipomas (AMLs) or cysts, or cardiac rhabdomyomas did not appear to be associated with a lower QoL.

Upon examining the impact of cognitive level on QoL, we identified a significant association between cognitive impairment and PedsQL scores (total score, *p* < 0.001; health and physical activity, *p* < 0.001; emotional states, *p* = 0.022; social life, *p* < 0.001; school, *p* < 0.001). When comparing groups, we found that patients with mild to severe ID had lower QoL than those with normal cognitive functioning. However, we observed no significant differences among patients with an average IQ and borderline intellectual functioning (BIF), nor among patients with different levels of ID (mild, moderate, and severe).

Regarding the association between neuropsychiatric symptoms, reported by parents using the TAND Checklist, and PedsQL scores, almost all symptoms were associated with lower PedsQL total scale scores, indicative of reduced QoL.

The complete statistics on the association between cognitive levels and neuropsychiatric symptoms, as assessed using the TAND Checklist, with PedsQL scores are provided (see **Supplementary Table 3**, **Supplementary Table 4**).

Analyzing the relationship between the PedsQL results and the ABAS-II and CBCL scores, we found a positive correlation between ABAS-II and PedsQL and a negative correlation between CBCL and PedsQL across all examined subscales. These results suggest that reduced adaptive functioning and greater behavioral problems are associated with a decreased QoL in patients with TSC. The complete statistics are reported in **Supplementary Table 5**.

#### 3.2.2 TSCQoL

By relating the clinical characteristics of TSC patients with the results obtained from the TSCQoL (**Table 3**), we found a statistically significant relationship between an increased economic and time commitment and the presence of cognitive impairment, ASD, sleep disorders, and epilepsy. These findings are consistent with the results obtained from the PedsQL.

**Table 3:** Association between clinical presentation (based on previous clinical evaluations, databases, and parent interviews) and family commitment in TSC patients.

TSC	Statistics	TSCQoL Money	TSCQoL Time	TSCQoL Days of work	TSCQoL Days of school
Cognitive impairment	Mann–Whitney U	851.5	893.5	748	700.5
	*p*-Value	**<0.001**	**<0.001**	0.003	0.018
ASD	Mann–Whitney U	934.5	968.5	747	751
	*p*-Value	**<0.001**	**<0.001**	0.007	0.006
ADHD	Mann–Whitney U	408.5	385	397	370
	*p*-Value	0.475	0.732	0.595	0.922
Sleep disorders	Mann–Whitney U	13.734	12.957	2.175	4.990
	*p*-Value	**<0.001**	**<0.001**	0.140	0.025
Other neuropsychiatric manifestations	Mann–Whitney U	679.5	624.5	573	524
	*p*-Value	0.024	0.123	0.411	0.875
Epilepsy	Mann–Whitney U	959.5	899.5	780.5	769
	*p*-Value	**<0.001**	**<0.001**	0.002	0.003
SEGA	Mann–Whitney U	415	448	348	492.5
	*p*-Value	0.441	0.183	0.750	0.040
Renal angiomyolipomas or cysts	Mann–Whitney U	469.5	491.5	469	473.5
	*p*-Value	0.581	0.372	0.343	0.535
Cardiac rhabdomyomas	Mann–Whitney U	445	430	439.5	427
	*p*-Value	0.097	0.151	0.115	0.167
Retinal phacomas	Mann–Whitney U	535.5	567.5	459	457
	*p*-Value	0.064	0.017	0.490	0.505

TSC, tuberous sclerosis complex; ASD, autism spectrum disorder; ADHD, attention deficit hyperactivity disorder; SEGA, subependymal giant cell astrocytoma; TSC QoL, TSC Quality of Life questionnaire.

p-Value adjusted after Bonferroni correction at 0.002.

Bold values indicate statistically significant results.

We extended our investigation into the impact of symptoms on families’ lives by performing a univariate logistic regression analysis (**Table 4**). We transformed the TSCQoL variables from continuous to dichotomous, identifying a cut-off that indicated a significant economic and time investment compared to minimal or no commitment.

**Table 4:** Association between clinical presentation (based on previous clinical evaluations, databases, and parent interviews) and family commitment in a univariate logistic regression in TSC patients.

TSC	Statistics	TSCQoL Money (>€100/month)	TSCQoL Time (>1 hour/day)	TSCQoL Days of work (>3 days/month)	TSCQoL Days of school (>3 days/month)
Cognitive impairment	B	1.089	1.191	0.561	0.402
	OR	2.972	3.290	1.752	1.494
	CI 95%	1.743–5.067	1.859–5.823	1.168–2.629	1.01–2.21
	*p*-Value	**<0.001**	**<0.001**	**0.007**	**0.044**
ASD	B	3.125	2.583	1.117	1.022
	OR	22.75	13.286	3.056	2.778
	CI 95%	6.116–84.618	3.357–52.576	1.116–8.365	0.992–7.78
	*p*-Value	**<0.001**	**<0.001**	**0.030**	0.052
ADHD	B	-0.232	0.223	-0.511	-0.527
	OR	0.793	1.25	0.6	0.59
	CI 95%	0.243–2.586	0.361–4.327	0.177–2.035	0.163–2.134
	*p*-Value	0.701	0.725	0.412	0.422
Sleep disorders	B	2.095	1.299	0.822	0.526
	OR	8.125	3.667	2.275	1.692
	CI 95%	2.349–28.107	1.235–10.883	0.818–6.327	0.606–4.73
	*p*-Value	**<0.001**	**0.019**	0.115	0.316
Other neuropsychiatric manifestations	B	0.624	0.307		
	OR	1.867	2.359		
	CI 95%	0.673–5.18	0.472–3.916		
	*p*-Value	0.231	0.57		
Epilepsy	B	2.808	1.477	1.582	0.999
	OR	16.571	4.379	4.865	2.715
	CI 95%	4.906–55.973	1.365–14.045	1.691–14.002	0.957–7.701
	*p*-Value	**<0.001**	**0.013**	**0.003**	0.060
Drug-resistant epilepsy	B	0.981		0.956	0.474
	OR	2.667		2.6	1.607
	CI 95%	0.417–17.046		0.627–10.786	0.414–6.24
	*p*-Value	0.300		0.188	0.493
SEGA	B	0.734	0.992	-0.201	1.511
	OR	2.083	2.698	0.818	4.533
	CI 95%	0.621–6.992	0.808–9.006	0.252–2.653	1.32–15.564
	*p*-Value	0.235	0.107	0.738	**0.016**
Renal angiomyolipomas or cysts	B	0.251	1.657	0.369	0.619
	OR	1.286	5.241	1.446	1.857
	CI 95%	0.434–3.808	1.08–25.439	0.479–4.36	0.571–6.046
	*p*-Value	0.650	**0.040**	0.513	0.304
Cardiac rhabdomyomas	B	0.813	1.123	0.773	0.39
	OR	2.255	3.074	2.167	1.477
	CI 95%	0.65–7.82	0.616–15.343	0.593–7.911	0.402–5.418
	*p*-Value	0.200	0.171	0.242	0.557
Retinal phacomas	B	1.34	1.041	-0.136	-0.01
	OR	3.818	2.833	0.873	0.99
	CI 95%	1.091–13.364	0.911–8.809	0.292–2.609	0.324–3.026
	*p*-Value	**0.036**	0.072	0.807	0.986

TSC, tuberous sclerosis complex; ASD, autism spectrum disorder; ADHD, attention deficit hyperactivity disorder; SEGA, subependymal giant cell astrocytoma; B, logistic regression coefficient; OR, odds ratio; CI, confidence interval; TSCQoL, TSC Quality of Life questionnaire.

Bold values indicate statistically significant results.

For the economic investment, we used a monthly expenditure of €100 as the cut-off. Higher economic expenditure was significantly associated with the presence of active epilepsy [odds ratio (OR) = 16.57, confidence interval (CI) 95% = 4.9–55.97, *p* < 0.001], cognitive impairment (OR = 2.97, CI 95% = 1.74–5.06, *p* < 0.001), ASD (OR = 22.75, CI 95% = 6.12–84.62, *p* < 0.001), and sleep disorders (OR = 8.13, CI 95% = 2.35–28.11, *p* < 0.001).

Regarding time spent by families dealing with TSC-related issues, we considered a commitment of more than 1 hour per day to be significant. A greater amount of time was once again significantly associated with the presence of active epilepsy (OR = 4.38, CI 95% = 1.37–14.05, *p* = 0.013), cognitive impairment (OR = 3.29, CI 95% = 1.86–5.82, *p* < 0.001), ASD (OR = 13.29, CI 95% = 3.36–52.58, *p* < 0.001), and sleep disorders (OR = 3.67, CI 95% = 1.24–10.88, *p* = 0.019).

Considering the number of work and school days lost, we defined a loss of more than 3 days per month as significant. A greater loss of workdays was associated with the presence of active epilepsy (OR = 4.87, CI 95% = 1.69–14, *p* = 0.030) and cognitive impairment (OR = 1.75, CI 95% = 1.17–2.63, *p* = 0.007). A greater loss of school days was significantly related to cognitive impairment (OR = 1.49, CI 95% = 1.01–2.21, *p* = 0.044) and SEGA (OR = 4.53, CI 95% = 1.32–15.56, *p* = 0.016).

Due to the significant associations between ASD, epilepsy, and increased economic investment, time spent, and days of work lost, we supplemented our analysis by including ASD and epilepsy as covariates in a multivariable logistic regression model (**Table 5**). Regarding economic investment, the relationship remains significant for both ASD (OR = 10.284, CI 95% = 2.44–43.38, *p* = 0.002) and epilepsy (OR = 6.41, CI 95% = 1.59–25.91, *p* = 0.009). Regarding time commitment, only ASD remains statistically significant (OR = 12.74, CI 95% = 2.41–67.42, *p* = 0.003). For days of work lost, only the presence of epilepsy remains statistically significant (OR = 4.03, CI 95% = 1.1–14.61, *p* = 0.034).

**Table 5:** Association between economic and time investment and loss of workdays with ASD and epilepsy as covariates in a multivariable logistic regression in TSC patients.

TSC	Statistics	TSCQoL Money (>€100/month)	TSCQoL Time (>1 hour/day)	TSCQoL Days of work (>3 days/ month)
Epilepsy	B	1.858	0.069	1.392
	OR	6.414	1.072	4.025
	CI 95%	1.587–25.914	0.222–5.172	1.108–14.614
	*p*-Value	**0.009**	0.931	**0.034**
ASD	B	2.331	2.545	0.32
	OR	10.284	12.743	1.377
	CI 95%	2.438–43.382	2.408–67.422	0.385–4.922
	*p*-Value	**0.002**	**0.003**	0.623

TSC, tuberous sclerosis complex; ASD, autism spectrum disorder; B, logistic regression coefficient; OR, odds ratio; CI, confidence interval; TSCQoL, TSC Quality of Life questionnaire.

Bold values indicate statistically significant results.

Relating to families’ concerns about their children’s conditions, an increase in families’ concern was observed for both their children’s current and future conditions associated with cognitive impairment, ASD, sleep disorders, and epilepsy. However, this association was not found for systemic alterations (such as SEGAs, renal AMLs or cysts, cardiac rhabdomyomas, and retinal phacomas) and for ADHD and other neuropsychiatric manifestations. The complete statistics are shown in **Supplementary Table 6**.

### 3.3 Comparison between TSC and ASD populations

A secondary objective of our study was to compare the QoL and disease burden of subjects with TSC and then with ASD secondary to TSC with those with idiopathic ASD.

#### 3.3.1 Neuropsychological tests

Patients with TSC exhibited better global and domain-specific adaptive functioning than patients with ASD (GAC, t = 2.582, *p* = 0.011; CAD, t = 2.445, *p* = 0.016; SAD, t = 3.414, *p* < 0.001; PAD, t = 2.145, *p* = 0.034).

Patients with TSC-related ASD had lower scores than those with idiopathic ASD in all areas, although the differences were not statistically significant after applying a *post-hoc* correction (GAC, t = 2.608, *p* = 0.011; CAD, t = 2.846, *p* = 0.005; SAD, t = 1.301, *p* = 0.119; PAD, t = 2.313, *p* = 0.023).

When analyzing behavioral difficulties, patients with TSC scored lower than those with ASD (total scale, t = 2.417, *p* = 0.017; internalizing, t = 3.375, *p* < 0.001; externalizing, t = 2.382, *p* = 0.019). However, patients with idiopathic ASD presented with more internalizing problems than those with TSC-related syndromic ASD, although this did not reach statistical significance after *post-hoc* correction (total scale, t = 0.391, *p* = 0.697; internalizing, 2.089, *p* = 0.039; externalizing, 0.094, *p* = 0.925). The *p*-value was adjusted to 0.004 after Bonferroni correction.

#### 3.3.2 PedsQL

Patients with TSC had similar overall QoL scores to the ASD population, as well as similar scores in the areas of health, physical activity, emotional states, and school. However, the ASD population had significantly lower scores in the area of social life (t = 2.928, *p* = 0.004) (**Table 6**).

**Table 6:** Comparison of PedsQL scores between TSC and ASD populations.

TSC and ASD comparison		N	Mean ± SD	t^*^	P-value
PedsQL Total	TSC	66	72.92 ± 19.955	1.805	0.073
	ASD	63	67.36 ± 14.478		
PedsQL Health and physical activity	TSC	66	77.72 ± 24.030	1.022	0.309
	ASD	63	86.55 ± 90.799		
PedsQL Emotional states	TSC	66	73.94 ± 20.931	1.990	0.049
	ASD	63	67.06 ± 18.131		
PedsQL Social life	TSC	66	72.35 ± 24.996	2.928	**0.004**
	ASD	63	60.24 ± 21.784		
PedsQL School	TSC	64	69.43 ± 25.060	0.725	0.470
	ASD	58	66.60 ± 16.834		

TSC, tuberous sclerosis complex; ASD, autism spectrum disorder; PedsQL, Pediatric Quality of Life Inventory; N, number of observations; SD, standard deviation.

^*^ Independent-samples t-test, with p-value adjusted using Bonferroni correction at 0.005.

Bold values indicate statistically significant results.

Patients with TSC-related ASD had lower overall QoL (*p* = 0.041) and poorer school functioning (*p* = 0.023) than children with idiopathic ASD. However, these differences were not statistically significant after applying the *post-hoc* correction (**Table 7**).

**Table 7:** Comparison of PedsQL scores between TSC-related ASD and ASD populations.

TSC-ASD and ASD comparison		N	Mean ± SD	t^*^	P-value
PedsQL Total	TSC-ASD	32	60.60 ± 16.921	2.074	0.041
	ASD	63	67.36 ± 14.478		
PedsQL Health and physical activity	TSC-ASD	32	60.17 ± 23.431	1.638	0.105
	ASD	63	86.55 ± 90.799		
PedsQL Emotional states	TSC-ASD	32	65.00 ± 19.797	0.493	0.623
	ASD	63	67.06 ± 18.131		
PedsQL Social life	TSC-ASD	32	57.81 ± 23.347	0.548	0.585
	ASD	63	60.24 ± 21.784		
PedsQL School	TSC-ASD	31	56.78 ± 22.740	2.322	0.023
	ASD	58	66.60 ± 16.834		

TSC-ASD, tuberous sclerosis complex-related autism spectrum disorder; ASD, autism spectrum disorder; PedsQL, Pediatric Quality of Life Inventory; N, number of observations; SD, standard deviation.

^*^ Independent-samples t-test, with p-value adjusted using Bonferroni correction at 0.005.

#### 3.3.3 TSCQoL

Using the chi-squared test, we compared the TSCQoL results between the entire TSC population and subjects with idiopathic ASD, as well as between TSC-related ASD patients and those with idiopathic ASD (see **Supplementary Table 7** and **Supplementary Table 8**).

Overall, 55% of patients with TSC had an economic investment of more than €100 per month, compared with 86% of subjects with idiopathic ASD (*p* < 0.001). However, when considering only the ASD subjects within the TSC population, this percentage increased to 82%, basically like the percentage seen in idiopathic ASD.

Additionally, 32% of TSC patients, 56% of TSC-ASD patients, and 51% of ASD subjects committed more than 1 hour per day. The idiopathic ASD population invested significantly more hours than the TSC population (*p* = 0.029); however, no significant differences were observed between the TSC-related ASD and the idiopathic ASD populations.

Regarding workdays lost, no significant differences were found among the three groups.

Finally, more than three school days per month were lost by 38% of TSC patients, 50% of TSC-ASD patients, and 29% of ASD subjects. This resulted in a significantly greater loss in the TSC-related syndromic ASD group than in the idiopathic ASD group (*p* = 0.039).

## 4 Discussion

Our analysis of data on the TSC-affected population revealed a significant reduction in QoL and an increased need for family support and resources.

Our findings align with previous literature, which has predominantly focused on adult populations. A 2020 systematic review analyzed 33 studies on disease burden in TSC. However, only 14 of these studies specifically addressed the individual burden and the indirect costs to families (10). The review revealed that neurological and psychiatric manifestations, particularly ID and drug-resistant epilepsy, significantly impact QoL (28–30). Additional studies have demonstrated that the burden on caregivers is closely linked to severe epilepsy and associated neuropsychiatric conditions (11, 14, 31).

A recent study (12) confirmed a major impact of epilepsy and neuropsychiatric manifestations on patients and their caregivers alike. Notably, a higher seizure frequency was associated with a greater disease burden, independent of neuropsychiatric factors. Another 2023 study by Skrobanski et al. revealed the impact of the condition on the work productivity and careers of caregivers (32).

In pediatric populations, although few studies are available, the literature consistently shows a reduction in QoL, which tends to worsen as patients transition into adolescence and adulthood (33). Specifically, QoL is lower in patients with a *TSC2* mutation, epilepsy, a longer disease course, high seizure frequency, ID, and TANDs (34). Caregivers of these patients also tend to experience higher levels of depressive symptoms (35).

A 2024 study (36) compared direct and indirect costs, as well as QoL, among adults with TSC-related epilepsy, idiopathic generalized epilepsy, and focal epilepsy in Germany. The study confirmed the significant economic costs and excessive QoL burden faced by patients with TSC.

Unlike previous studies, our research focused exclusively on a homogeneous pediatric cohort in terms of age, intellectual functioning, and other neuropsychiatric comorbidities. This allowed for a detailed characterization of QoL in this group. Our analysis revealed a reduction in QoL with significant variability: 50% of patients had at least acceptable PedsQL scores, while the remaining patients had QoL results ranging from poor to very poor.

We found that lower adaptive functioning and higher levels of internalizing and externalizing behavioral disorders were closely related to QoL scores. There were significant associations between QoL and factors such as active epilepsy, ID ranging from mild to severe, ASD, and sleep disorders. These findings are consistent with previous literature (11, 12, 14, 30, 31). Thus, neuropsychiatric impairment appears central in determining the overall impact of TSC on patients and their families. Interestingly, potentially severe systemic conditions, such as renal AMLs, cardiac rhabdomyomas, and SEGAs, did not show an association with reduced QoL as assessed using the PedsQL scale.

A key innovation of our study was the development of the TSCQoL, a specific QoL scale tailored to the TSC population. The scale was easy to administer, while it effectively highlighted clinical factors that most impact QoL, such as epilepsy and neuropsychiatric manifestations, consistent with PedsQL results. Furthermore, the TSCQoL scale revealed that families of patients with poorer QoL experienced greater time and financial burdens. However, we acknowledge that this instrument has not yet been validated, and our findings should be interpreted with caution. Future research is needed to formally assess its psychometric properties and establish its reliability and validity.

Both the TSCQoL and the TAND Checklist provided valuable insights into family stress and concerns. Over 60% of TSC families reported being highly worried about their child’s current and future conditions. Neuropsychiatric, neurological, and renal disorders were identified as the main sources of concern. The TAND Checklist also revealed significant family and parental stress, with two-thirds of respondents seeking support from the healthcare system. As highlighted both by the literature and by our findings, which emphasize the critical role of neuropsychiatric issues in determining QoL, it is concerning that these symptoms are often only partially evaluated in patients with TSC, as indicated by the TOSCA study (37).

To better understand the impact of the syndromic component versus ASD on QoL and disease burden, we compared the TSC population with a cohort of patients with idiopathic ASD. In the idiopathic ASD group, we observed greater behavioral difficulties, more impaired adaptive functioning, and a poorer QoL in the social domain compared to those of the TSC population. These patients also required more time and financial resources from their families, leading to higher levels of family stress. However, there were no significant differences in overall QoL between the TSC and idiopathic ASD groups.

When we compared patients with ASD secondary to TSC with those with idiopathic ASD, we found that the syndromic ASD group had worse overall QoL and adaptive functioning and experienced a higher number of missed school days.

Our study clearly shows that these populations, especially those with ASD, require substantial economic resources. Considering the average gross annual salary in Italy, which was approximately €35,995 in 2022 according to the Italian National Institute of Statistics (38), it becomes evident that the financial burden could be unsustainable for many families.

Another relevant finding is the significant number of workdays lost. Approximately one-half of the families in our study reported losing three or more workdays per month. This suggests that many families regularly exceed the leave entitlements provided by Law 104/1992 in Italy, which establishes the rights and assistance of people with disabilities and grants up to 3 days off per month (36 days per year) for severe disabilities.

Our study has some limitations. First, the used questionnaires rely on reports from parents or caregivers, without directly involving the patients. This could lead to discrepancies between the parents’ perceptions and the patient’s actual perspectives and experiences. However, direct involvement of patients is often not feasible in cases of ID or ASD due to communication barriers.

Another limitation of this study is the lack of an assessment of the severity of autistic symptoms through direct, standardized evaluations, such as the ADOS test, or questionnaires administered to parents. Notably, QoL in ASD populations correlates with varying symptom intensity, which in turn relates to adaptive functioning levels. Future studies should incorporate standardized ASD severity assessments alongside cognitive measures to better delineate their independent contributions to adaptive functioning and QoL. Another point to consider is the possible influence of a selection bias on our results. The children included in the study are from two tertiary referral hospitals, which may have led to the selection of subjects with more severe clinical features than the entire ASD or TSC populations. Furthermore, the study exclusively analyzed an Italian population. Leave entitlements for caring for people with disabilities may vary according to country-specific legislation. This could result in different illness impacts among individuals in different countries. Therefore, the disease burden detected in our sample may differ from that of a similar population in another country. Finally, this work did not address the socioeconomic status of families, which can influence both the perception of QoL and the resources available to cope with the disease burden.

## 5 Conclusions

This study confirmed that individuals affected by TSC have a reduced QoL, and there is a strong association between QoL and neuropsychiatric and neurological manifestations, such as cognitive impairment, ASD, sleep disorders, and epilepsy. Additionally, our results showed that families face significant economic and time burdens when managing the disease. Despite some level of state assistance in Italy, these investments far exceed the support currently provided by public services, leaving most of the burden on families. Available resources are often insufficient to fully meet these patients’ complex needs, highlighting a gap in support that demands attention.

In our study, ASD emerged as one of the most significant factors contributing to the disease burden. However, comparing TSC-related ASD patients with idiopathic ASD patients revealed that the syndromic condition has an even greater impact on disease burden and QoL than ASD alone.

Considering both the literature and our findings, which underscore the critical role of neuropsychiatric issues in determining QoL, it is concerning that these symptoms are often only partially evaluated in patients with TSC. The routine use of the TAND Checklist, followed by a thorough assessment of developmental, emotional, and behavioral problems, should be an integral part of the clinical follow-up of TSC patients. Furthermore, TSC’s multisystem nature affects not only physical health but also the well-being and perceived QoL of both patients and their families.

## Data Availability

The raw data supporting the conclusions of this article will be made available by the authors, without undue reservation.
