# Supplementary material for: Quality of life and disease burden in tuberous sclerosis and comparison with the population with idiopathic autism spectrum disorder: an investigation conducted through questionnaires and clinical data collection in the pediatric population
Source: Front Psychiatry. 2026 Jan 14;16:1730160. doi: 10.3389/fpsyt.2025.1730160 (PMC12847237; doi:10.3389/fpsyt.2025.1730160)
Supplement: Supplementary file 1 [file DataSheet1.pdf]

TSC Quality of Life questionnaire

The TSC Quality of Life questionnaire is a parent-administered questionnaire developed to assess the TSC-related emotional, economic, and time investments.

Regarding the emotional aspect, caregivers were asked to assign a score from 0 (indicating no concern) to 5 (indicating extreme concern), regarding neurological, psychiatric and systemic issues related to TSC. Specifically, in our analysis, we considered scores of 4 and 5 as high levels of concern.

Furthermore, we asked families to estimate the economic investment required to meet their childs healthcare needs, choosing from several ranges per month (none, below €100, between €100 and €300, between €300 and €500, above €500). Regarding time commitment, we considered the number of hours required for the family or patient to actively address the challenges arising from TSC (e.g., preparation and administration of medications, rehabilitation therapy, medical appointments, diagnostic investigations); ranges to be selected were: below 1 hour per week, between 1 and 3 hours per week, between 4 and 5 hours per week, between 1 and 2 hours per day, and 3 hours or above per day. Additionally, we asked families to report the number of days they and the patients were unable to participate in work and/or school activities due to the difficulties associated with the underlying condition (none, between 1 and 2 days, between 3 and 5 days, between 6 and 10 days, or above 10 days per month).
