# Supplementary material for: Quality of life and disease burden in tuberous sclerosis and comparison with the population with idiopathic autism spectrum disorder: an investigation conducted through questionnaires and clinical data collection in the pediatric population
Source: Front Psychiatry. 2026 Jan 14;16:1730160. doi: 10.3389/fpsyt.2025.1730160 (PMC12847237; doi:10.3389/fpsyt.2025.1730160)
Supplement: Supplementary file 2 [file DataSheet2.pdf]

SUPPLEMENTARY TABLE 1 PedsQL results, distribution and descriptive statistics of scores in TSC patients.

TSC	PedsQL Total	PedsQL Health and Physical Activity	PedsQL Emotional States	PedsQL Social Life	PedsQL School
	N (%)	N (%)	N (%)	N (%)	N (%)
Optimal	26 (39%)	27 (41%)	30 (45%)	29 (44%)	27 (42%)
Acceptable	7 (11%)	3 (5%)	10 (15%)	9 (14%)	12 (19%)
Minor impairment	5 (8%)	10 (15%)	0 (0%)	3 (5%)	2 (3%)
Moderate impairment	8 (12%)	8 (12%)	6 (9%)	3 (5%)	6 (9%)
Severe impairment	20 (30%)	18 (27%)	20 (30%)	22 (33%)	17 (27%)
Total	66 (100%)	66 (100%)	66 (100%)	66 (100%)	66 (100%)
	N	N	N	N	N
Mean	72.9	74.7	73.9	72.4	69.4
Median	75.45	82.85	80	80	70
Standard Deviation	20	24	20.9	25	25.1
Minimum value	28.8	15.6	15	10	0
Maximum value	100	100	100	100	100

Abbreviations: TSC: Tuberous Sclerosis Complex; PedsQL: Pediatric Quality of Life Inventory; N: number of observations.

SUPPLEMENTARY TABLE 2 TSCQoL results, impact of disease on TSC families.

TSC	N (%)	N (%)	N (%)	N (%)	N (%)
Economic investment	None	<€100/month	€100-300 /month	€300-500 /month	>€500/month
	17 (26%)	13 (20%)	14 (21%)	9 (14%)	13 (20%)
Time commitment	<1h/week	1-3 h/week	4-5 h/week	1-2 h/day	>3 h/day
	26 (39%)	10 (15%)	9 (14%)	7 (11%)	14 (21%)
Days lost	None	1-2 days	3-5 days	6-10 days	>10 days
Work	19 (29%)	17 (26%)	14 (21%)	9 (14%)	7 (11%)
School	18 (27%)	23 (35%)	14 (21%)	7 (11%)	4 (6%)

Abbreviations: TSC: Tuberous Sclerosis Complex; N: number of observations.

SUPPLEMENTARY TABLE 3 Association between cognitive levels and PedsQL in TSC patients.

TSC		PedsQL Total	PedsQL Health and Physical Activity	PedsQL Emotional States	PedsQL Social Life	PedsQL School
Cognitive impairment	Kruskal-Wallis	28.949	28.055	11.394	30.190	21.938
	p-value	**<0.001**	**<0.001**	**0.022**	**<0.001**	**<0.001**
No cognitive impairment vs. BIF	Dunn's test	6.359	7.965	0.222	4.519	8.004
	p-value	1	1	1	1	1
No cognitive impairment vs. Mild ID	Dunn's test	20.426	20.190	11.222	20.477	19.862
	p-value	0.220	**0.023**	0.899	**0.019**	0.210
No cognitive impairment vs. Moderate ID	Dunn's test	27.081	26.208	19.044	27.447	22.577
	p-value	**<0.001**	**<0.001**	**0.024**	**<0.001**	**0.030**
No cognitive impairment vs. Severe ID	Dunn's test	40.426	41.315	13.389	39.352	33.987
	p-value	**0.005**	**0.004**	1	**0.007**	**0.026**
BIF vs. Mild ID	Dunn's test	14.067	12.225	11	15.958	11.858
	p-value	0.869	1	1	0.498	1
BIF vs. Moderate ID	Dunn's test	20.721	18.243	18.821	22.929	14.573
	p-value	0.910	0.212	0.171	**0.036**	0.610
BIF vs. Severe ID	Dunn's test	34.067	33.350	13.167	34.833	25.983
	p-value	0.070	0.080	1	0.053	0.328
Mild ID vs. Moderate ID	Dunn's test	6.655	6.018	7.821	6.970	2.715
	p-value	1	1	1	1	1
Mild ID vs. Severe ID	Dunn's test	20	21.122	2.167	18.875	14.125
	p-value	1	0.869	1	1	1
Moderate ID vs. Severe ID	Dunn's test	13.345	15.107	0.222	11.905	11.410
	p-value	1	1	1	1	1

Abbreviations: TSC: Tuberous Sclerosis Complex; vs.: versus; BIF: Borderline Intellectual Functioning; ID: Intellectual Disability; PedsQL: Pediatric Quality of Life Inventory.

SUPPLEMENTARY TABLE 4 Association between neuropsychiatric symptoms assessed by TAND Check-List and PedsQL scores in TSC patients.

TSC		PedsQL Total	PedsQL Health and Physical Activity	PedsQL Emotional States	PedsQL Social Life	PedsQL School
Anxiety	Mann-Whitney U	310	433.5	227	332.5	327
	p-value	0.018	0.490	**<0.001**	0.037	0.055
Mood alterations	Mann-Whitney U	175.5	232	169	188	266
	p-value	**<0.001**	**<0.001**	**<0.001**	**<0.001**	0.004
Aggression and self-harm	Mann-Whitney U	251.5	258.5	299	307.5	260
	p-value	0.002	0.003	0.016	0.022	0.009
Angry outbursts	Mann-Whitney U	234	301	264	274	262
	p-value	**<0.001**	0.002	**<0.001**	**<0.001**	**<0.001**
Social relationship difficulties	Mann-Whitney U	115.5	156	143.5	142	190.5
	p-value	**<0.001**	**<0.001**	**<0.001**	**<0.001**	**<0.001**
Rigid and repetitive behaviors	Mann-Whitney U	186	218	340	206	170
	p-value	**<0.001**	**<0.001**	0.010	**<0.001**	**<0.001**
Hyperactivity-impulsivity	Mann-Whitney U	306	337.5	363	334.5	309
	p-value	0.002	0.008	0.019	0.007	0.006
Deficit in attention and memory	Mann-Whitney U	90	132	243	117.5	67.5
	p-value	**<0.001**	**<0.001**	0.006	**<0.001**	**<0.001**
Deficit in executive functions	Mann-Whitney U	156	194	271	189.5	167
	p-value	**<0.001**	**<0.001**	**<0.001**	**<0.001**	**<0.001**
Eating disorders	Mann-Whitney U	146.5	188.5	188.5	166.5	132.5
	p-value	**<0.001**	**0.001**	**0.001**	**<0.001**	**<0.001**

Abbreviations: TSC: Tuberous Sclerosis Complex; PedsQL: Pediatric Quality of Life Inventory.
p-value adjusted after Bonferroni correction at 0.002.

SUPPLEMENTARY TABLE 5 Correlation between ABAS-II and CBCL with PedsQL scores in TSC patients.

TSC		PedsQL Total	PedsQL Health and Physical Activity	PedsQL Emotional States	PedsQL Social Life	PedsQL School
ABAS-II GAC	Spearman's rho	0.800	0.800	0.535	0.715	0.739
	p-value	**<0.001**	**<0.001**	**<0.001**	**<0.001**	**<0.001**
ABAS-II CAD	Spearman's rho	0.733	0.763	0.458	0.661	0.695
	p-value	**<0.001**	**<0.001**	**<0.001**	**<0.001**	**<0.001**
ABAS-II SAD	Spearman's rho	0.693	0.716	0.455	0.567	0.681
	p-value	**<0.001**	**<0.001**	**<0.001**	**<0.001**	**<0.001**
ABAS-II PAD	Spearman's rho	0.781	0.754	0.551	0.725	0.692
	p-value	**<0.001**	**<0.001**	**<0.001**	**<0.001**	**<0.001**
CBCL Total	Spearman's rho	-0.763	-0.684	-0.659	-0.660	-0.662
	p-value	**<0.001**	**<0.001**	**<0.001**	**<0.001**	**<0.001**
CBCL Internalizing	Spearman's rho	-0.553	-0.393	-0.647	-0.446	-0.446
	p-value	**<0.001**	**<0.001**	**<0.001**	**<0.001**	**<0.001**
CBCL Externalizing	Spearman's rho	-0.528	-0.498	-0.472	-0.427	-0.432
	p-value	**<0.001**	**<0.001**	**<0.001**	**<0.001**	**<0.001**

Abbreviations: TSC: Tuberous Sclerosis Complex; ABAS-II: Adaptive Behavior Assessment System – Second Edition; GAC: General Adaptive Composite; CAD: Conceptual Adaptive Domain; SAD: Social Adaptive Domain; PAD: Practical Adaptive Domain; CBCL: Child Behavior Checklist; PedsQL: Pediatric Quality of Life Inventory.

SUPPLEMENTARY TABLE 6 Association between clinical presentation and families' concern in TSC patients.

TSC		TSCQoL Total concern about current condition	TSCQoL Total concern about future condition
Cognitive impairment	Mann-Whitney U	796.5	756
	p-value	**<0.001**	**0.001**
ASD	Mann-Whitney U	791.5	758
	p-value	**0.001**	**0.003**
ADHD	Mann-Whitney U	326	305.5
	p-value	0.534	0.318
Sleep disorders	Mann-Whitney U	724	712.5
	p-value	**0.002**	**0.002**
Other neuropsychiatric manifestations	Mann-Whitney U	529.5	544
	p-value	0.815	0.650
Epilepsy	Mann-Whitney U	828.5	835
	p-value	**<0.001**	**<0.001**
SEGA	Mann-Whitney U	384.5	394.5
	p-value	0.779	0.644
Renal angiomyolipomas or cysts	Mann-Whitney U	508.5	455
	p-value	0.250	0.719
Cardiac rhabdomyomas	Mann-Whitney U	366	428.5
	p-value	0.718	0.141
Retinal phacomas	Mann-Whitney U	528	513
	p-value	0.075	0.105

Abbreviations: TSC: Tuberous Sclerosis Complex; ASD: Autism Spectrum Disorder; ADHD: Attention Deficit Hyperactivity Disorder; SEGA: Subependymal giant cell astrocytomas; TSCQoL: TSC Quality of Life questionnaire.
p-value adjusted after Bonferroni correction at 0.004.

SUPPLEMENTARY TABLE 7 Comparison of TSCQoL scores between TSC and ASD populations.

TSC and ASD Comparison		N	%	p-value*
TSCQoL Money >€100/month	TSC	36/66	55%	**<0.001**
	ASD	54/63	86%	
TSCQoL Time >1 hour/day	TSC	21/66	32%	**0.029**
	ASD	32/63	51%	
TSCQoL Work >3 days/month	TSC	30/66	46%	0.948
	ASD	29/63	47%	
TSCQoL School >3 days/month	TSC	25/66	38%	0.262
	ASD	18/63	29%	

Abbreviations: TSC: Tuberous Sclerosis Complex; ASD: Autism Spectrum Disorder; TSCQoL: TSC Quality of Life questionnaire.
*Chi squared test.

SUPPLEMENTARY TABLE 8 Comparison of TSCQoL scores between TSC-related ASD and ASD populations.

TSC-ASD and ASD Comparison		N	%	p-value*
TSCQoL Money >€100/month	TSC-ASD	28/32	82%	0.811
	ASD	54/63	86%	
TSCQoL Time >1 hour/day	TSC-ASD	18/32	56%	0.615
	ASD	32/63	51%	
TSCQoL Work >3 days/month	TSC-ASD	19/32	60%	0.219
	ASD	29/63	47%	
TSCQoL School >3 days/month	TSC-ASD	16/32	50%	**0.039**
	ASD	18/63	29%	

Abbreviations: TSC-ASD: Tuberous Sclerosis Complex-related Autism Spectrum Disorder; ASD: idiopathic Autism Spectrum Disorder; TSCQoL: TSC Quality of Life questionnaire.
*Chi squared test.
